# Resectable Pancreatic Adenocarcinomas following Neoadjuvant Chemotherapy with Gemcitabine Plus S-1: Two Case Reports of Opposite Oncological Outcomes

**DOI:** 10.70352/scrj.cr.25-0156

**Published:** 2025-08-08

**Authors:** Masanobu Taguchi, Hideki Sasanuma, Masayuki Shinoda, Yoshiyuki Meguro, Kazue Morishima, Hideyo Miyato, Hideyuki Ohzawa, Kazuhiro Endo, Naoki Sano, Hirotoshi Kawata, Noriyoshi Fukushima, Yasunaru Sakuma, Hironori Yamaguchi, Joji Kitayama, Naohiro Sata

**Affiliations:** 1 Department of Surgery, Jichi Medical University School of Medicine, Shimotsuke, Tochigi, Japan; 2 Department of Pathology, Jichi Medical University, Shimotsuke, Tochigi, Japan

**Keywords:** resectable pancreatic adenocarcinoma, neoadjuvant chemotherapy, gemcitabine, S-1, pathological complete response, pathological splenic vein involvement, early recurrence

## Abstract

**INTRODUCTION:**

Neoadjuvant gemcitabine plus S-1 (GS) therapy for resectable pancreatic cancer has been shown to prolong overall survival significantly compared with upfront surgery. Herein, we report two opposite cases of patients with resectable pancreatic cancer who underwent distal pancreatectomy after neoadjuvant GS therapy.

**CASE PRESENTATION:**

In Case 1, a 49-year-old female with a 12 mm tumor in the pancreatic body (cT1N0M0, cStage IA, union for international cancer control [UICC] 8th edition) underwent two courses of neoadjuvant GS therapy followed by an open distal pancreatectomy. Pathological examination revealed no residual cancer and the patient was diagnosed with a pathological complete response (pCR) without recurrence 31 months after surgery. However, in Case 2, a 74-year-old male with a 12 mm tumor in the pancreatic body (cT1N0M0, cStage IA, UICC 8th edition) also underwent two courses of neoadjuvant GS therapy, and then a laparoscopic distal pancreatectomy was performed. Pathological examination showed invasive pancreatic ductal adenocarcinoma with a 20 mm tumor. The tumor exhibited invasion into the lumen of the splenic vein and retroperitoneal tissue (ypT1N0M0, ypStage IA, UICC 8th edition). Adjuvant chemotherapy with S-1 was started, but 4 months postoperatively, a significant rise in serum CA19-9 levels was observed with multiple hepatic metastases and portal venous tumor thrombus. Gemcitabine plus nab-paclitaxel (GnP) therapy was started, however, the tumor progressed rapidly. The patient died 6 months after surgery.

**CONCLUSIONS:**

Neoadjuvant GS therapy is potentially expected to have a significant therapeutic effect as the pCR. Nevertheless, even after surgical resection, some patients still exhibit extremely poor prognosis. Therefore, it is necessary to clarify their clinical characteristics.

## Abbreviations

CA19-9
: carbohydrate antigen 19-9
CEA
: carcinoembryonic antigen
dCK
: deoxycytidine kinase
DNA
: deoxyribonucleic acid
EUS-FNA
: endoscopic ultrasound-fine needle aspiration
FOLFIRINOX
: oxaliplatin, leucovorin, irinotecan, plus 5-fluorouracil
GnP
: gemcitabine plus nab-paclitaxel
GS
: gemcitabine plus S-1
hENT1
: human equilibrative nucleoside transporter 1
pCR
: pathological complete response
RECIST
: response evaluation criteria in solid tumors
RRM1
: ribonucleotide reductase M1
UICC
: union for international cancer control

## INTRODUCTION

The Prep-02/JSAP-05 trial showed that median overall survival for patients receiving neoadjuvant GS therapy for resectable pancreatic cancer was 37.0 months, significantly better compared to 26.6 months in the upfront surgery group.^1)^ The concept of neoadjuvant GS therapy is to improve R0 resection rate by downstaging the primary tumor, and to suppress micrometastasis early after diagnosis. However, there are not sufficient reports elucidating the mechanism of why this treatment prolongs survival. Furthermore, the impact of the histological response of neoadjuvant GS therapy on survival remains incompletely understood. In general, in clinical trials, patients with extremely poor prognosis are not individually reported, and thus, their clinical characteristics remain unclear.

The present study describes two opposite cases of resectable pancreatic cancer treated with neoadjuvant GS therapy followed by distal pancreatectomy. One case achieved a pCR and with no recurrence, while the other suffered early recurrence after surgery and rapid disease progression resulting in death.

## CASE PRESENTATION

**Fig. 1:**
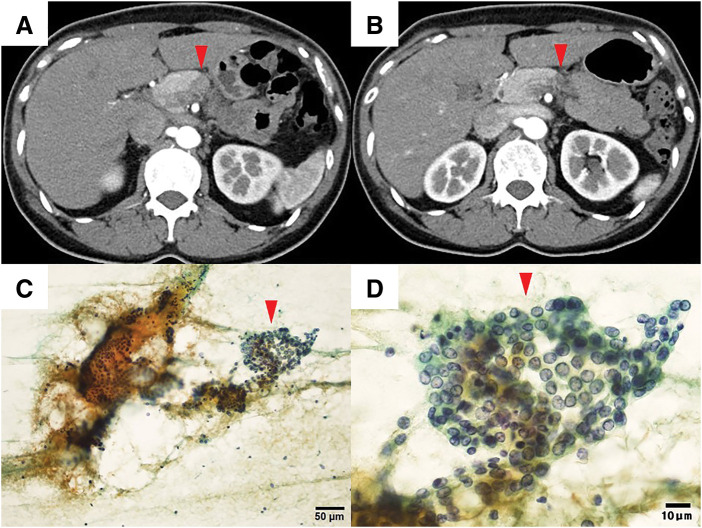
CT images and cytological findings of Case 1 (**A**) 12 mm hypovascular tumor observed in the pancreatic body (arrowhead) before neoadjuvant GS therapy. (**B**) Tumor reduced in size to 5 mm (arrowhead) after neoadjuvant GS therapy. (**C**, **D**) A loosely cohesive cell cluster with nuclear irregularities and fine chromatin was observed, leading to a diagnosis of adenocarcinoma (arrowhead). (Papanicolaou staining, ×200; **C**) (Papanicolaou staining, ×400; **D**). GS, gemcitabine plus S-1

**Fig. 2:**
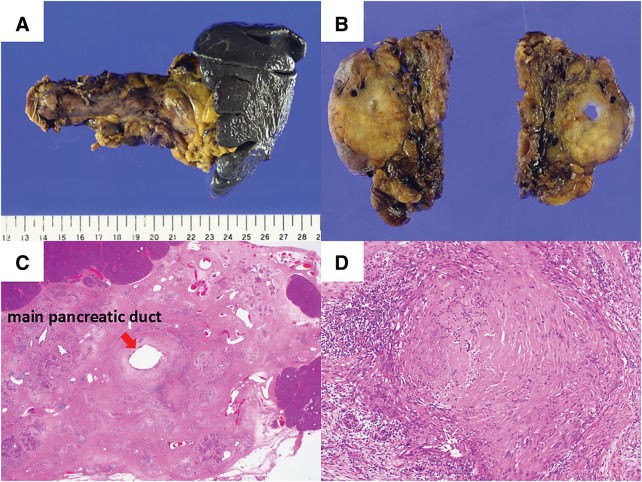
Pathological findings of resected specimen in Case 1 (**A**, **B**) Macroscopically, a 5 mm white-colored area localized to the main pancreatic duct 15 mm from the pancreatic margin. Distally, the main pancreatic duct was dilated, and pancreatic parenchyma appeared atrophic. (**C**, **D**) No residual cancer was observed in the white-colored area surrounding the main pancreatic duct, confirming diagnosis of pathological complete response. (Hematoxylin and eosin staining, ×12.5; **C**) (Hematoxylin and eosin staining, ×100; **D**).

Case 1: A 49-year-old female was referred to our hospital with elevated serum pancreatic enzyme level from a routine health checkup, and imaging studies suggested possibility of pancreatic body cancer. Contrast-enhanced CT revealed a 12 mm hypovascular tumor in the pancreatic body (**Fig. 1A**), with no evidence of portal venous or arterial system invasion nor obvious lymph node or distant metastasis. EUS-FNA was performed on the lesion, and cytological findings revealed the adenocarcinoma (**Fig. 1C**, **1D**). Contrast-enhanced MRI showed no liver metastasis. Tumor markers were CEA 1.2 ng/mL and CA19-9 14 U/mL. The patient was diagnosed with resectable pancreatic body cancer (cT1cN0M0, cStage IA, UICC 8th edition, and planned surgery after neoadjuvant GS therapy. Although neoadjuvant GS therapy was delayed and the dose was reduced due to leukopenia and neutropenia, two courses were completed as planned. Contrast-enhanced CT revealed a reduction in tumor size to 5 mm (**Fig. 1B**), and treatment response was assessed as Partial Response using the RECIST v1.1. CEA was 0.8 ng/mL, and CA19-9 was 10 U/mL, both within normal range. Contrast-enhanced MRI again showed no liver metastasis. An open distal pancreatectomy was performed 74 days after treatment initiation. No postoperative complications were observed, and the patient was discharged on POD 15. Macroscopic findings of the resected specimen revealed a 5 mm white area around the main pancreatic duct, located 15 mm from the pancreatic margin, with dilatation of the duct and atrophy of the pancreatic parenchyma distal to this area (**Fig. 2A**, **2B**). Microscopic findings of this white area showed fibrosis and atrophy of acinar cells, with no evidence of residual cancer, and the patient was diagnosed as a pCR (**Fig. 2C**, **2D**). No lymph node metastasis was observed, and peritoneal washing cytology was negative. Adjuvant chemotherapy with S-1 was initiated on POD 34. The patient remains alive with no recurrence 31 months after surgery.

**Fig. 3:**
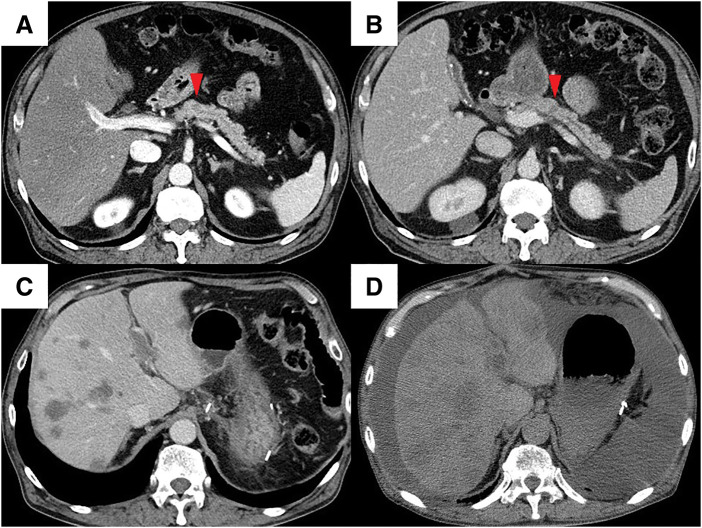
CT images of Case 2 (**A**) 12 mm hypovascular tumor observed in the pancreatic body (arrowhead) before neoadjuvant GS therapy. (**B**) Tumor remained 11 mm in diameter, with no change in size (arrowhead) after neoadjuvant GS therapy. (**C**) Multiple hepatic metastases and portal vein tumor thrombus were observed on POD 125. (**D**) Increased hepatic metastasis and ascites were shown on POD 176. GS, gemcitabine plus S-1

**Fig. 4:**
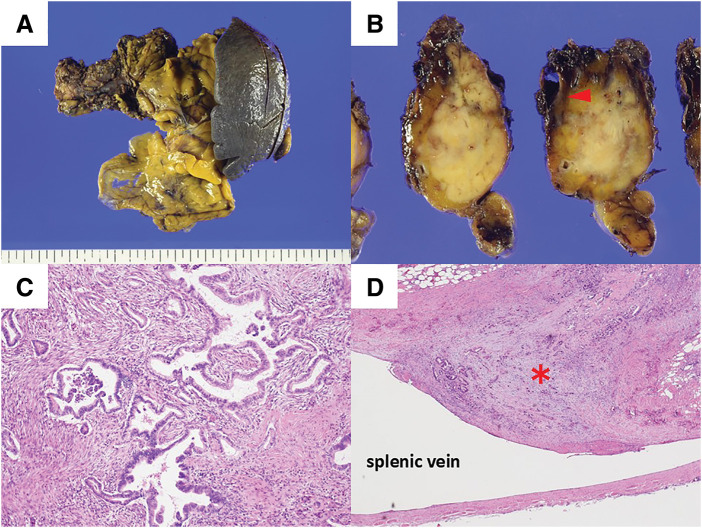
Pathological findings of the resected specimen in Case 2 (**A**, **B**) Macroscopically, a 20 mm tumor in the pancreatic body was observed with invasion into the lumen of the splenic vein (arrowhead). (**C**) The majority of the tumor consisted of well-differentiated adenocarcinoma, with areas of moderately differentiated adenocarcinoma, leading to the diagnosis of invasive pancreatic ductal adenocarcinoma. (Hematoxylin and eosin staining, ×100). (**D**) Tumor cell invasion into the lumen of the splenic vein (asterisk). (Hematoxylin and eosin staining, ×40).

Case 2: An abdominal ultrasonography before endoscopic treatment for early gastric cancer revealed a mass in the pancreatic body of a 74-year-old male. Contrast-enhanced CT revealed a 12 mm hypovascular tumor with no evidence of invasion into the portal or arterial systems including the splenic vein and artery, and no clear lymph node or distant metastasis (**Fig. 3A**). EUS-FNA confirmed the adenocarcinoma. Contrast-enhanced MRI and positron emission tomography revealed no distant metastasis. Tumor markers were CEA 0.8 ng/mL and CA19-9 23 U/mL. The patient was diagnosed with resectable pancreatic body cancer (cT1N0M0, cStage IA, UICC 8th edition), and planned surgery after neoadjuvant GS therapy. Although GS therapy was delayed due to leukopenia and neutropenia, the planned two courses were completed. Contrast-enhanced CT revealed the tumor remained 11 mm in size (**Fig. 3B**), with no evidence of radiological splenic vein involvement, and treatment response categorized as Stable Disease using RECIST v1.1. CEA was 0.9 ng/mL, and CA19-9 was 16 U/mL, both within normal range. Contrast-enhanced MRI again showed no liver metastasis. A laparoscopic distal pancreatectomy was performed 66 days after treatment initiation. Postoperatively, the patient developed a Grade II (Clavien-Dindo classification v2.0) and Grade B pancreatic fistula (International Study Group of Pancreatic Surgery, 2016 version), but his condition improved conservatively and he was discharged on POD 15. Macroscopic findings of the resected specimen revealed a 20 mm tumor in the pancreatic body, with invasion into the lumen of the splenic vein (**Fig. 4A**, **4B**). Microscopic findings confirmed invasive pancreatic ductal adenocarcinoma, predominantly well-differentiated with some areas of moderately differentiated adenocarcinoma (ypT1N0M0, ypStage IA, UICC 8th edition) (**Fig. 4C**). Further microscopic findings showed tumor cell invasion into the splenic vein lumen (**Fig. 4D**), as well as invasion into retroperitoneal tissue. The histological response was graded as Grade IIa (Evans classification). Resection margins and peritoneal washing cytology were both negative. Adjuvant chemotherapy with S-1 was started on POD 34. The serum CA19-9 level mildly increased to 66 U/mL on POD 76, and increased substantially to 1407 U/mL on POD 125. Contrast-enhanced CT revealed multiple liver metastases and portal venous tumor thrombus (**Fig. 3C**). Adjuvant S-1 therapy was discontinued, and GnP therapy was initiated on POD 145. However, the patient developed cholangitis and decline in performance status, preventing further chemotherapy. The tumor progressed rapidly, and CT showed increased liver metastasis and ascites on POD 176 (**Fig. 3D**). The patient died 6 months after surgery.

## DISCUSSION

Both cases were cStage IA (UICC 8th edition) pancreatic body cancer that had successfully completed two planned courses of neoadjuvant GS therapy, achieved R0 resection, and subsequently treated with adjuvant chemotherapy, demonstrating that multidisciplinary treatment proceeded smoothly. Case 1 is a very rare case which obtained pCR following two courses of neoadjuvant GS therapy without radiotherapy. The patient is alive without recurrence 31 months after surgery. By contrast, Case 2 experienced early postoperative liver metastasis that progressed rapidly leading to death, the opposite outcome.

The GEST study showed that the response rate of GS therapy for unresectable pancreatic cancer was significantly better compared with gemcitabine alone (29.3% vs. 13.3%, *P* < 0.001), demonstrating the high antitumor effect of GS therapy.^2)^ Furthermore, in the Prep-02/JSAP-05 trial targeting resectable pancreatic cancer patients, the neoadjuvant GS therapy group showed significantly better overall survival compared with the upfront surgery group.^1)^ This suggests that GS therapy as neoadjuvant chemotherapy could be a promising strategy for improving survival of patients with resectable pancreatic cancer. On the other hand, achieving a pCR with GS therapy alone is very rare. In the Prep-02/JSAP-05 trial, histological evaluation was performed on 111 cases in the neoadjuvant GS group, but no pCR cases were observed.^1)^ Similarly, in the randomized trial (JASPAC 04), which investigated the therapeutic efficacy of neoadjuvant GS therapy and chemoradiotherapy for resectable pancreatic cancer, no pCR cases were found among the 44 cases in the neoadjuvant GS group.^3)^ Furthermore, in two retrospective studies evaluating the effects of neoadjuvant GS therapy, no pCR cases were identified.^4,5)^ PubMed search with the keywords “pancreatic cancer,” “gemcitabine,” “S-1,” and “pathological complete response” revealed only three reports for pCR after neoadjuvant GS therapy without radiotherapy.^6–8)^ Currently, the number of cases is extremely limited, however, neoadjuvant GS therapy seems to have the potential effect of pCR. Further studies are required to elucidate the clinical significance of pCR following neoadjuvant GS therapy.

Systematic reviews showed that the pCR rate in patients with pancreatic cancer is very low at 4%.^9)^ Recently, the reported prognosis of pCR patients is favorable, and addition of radiotherapy may increase the pCR rate. Cloyd et al. analyzed 7902 pancreatic cancer patients from the national cancer clinical database that underwent resection after neoadjuvant therapy.^10)^ Median overall survival of pCR patients was significantly better compared with non-pCR patients (76.6 vs. 26.6 months, *P* < 0.001), with longer duration of neoadjuvant therapy and preoperative radiotherapy identified as independent factors associated with pCR. Stoop et al.’s multicenter retrospective observational study analyzing 1758 pancreatic cancer patients^11)^ revealed that median overall survival of pCR patients was significantly better than non-pCR patients (not reached vs. 31.0 months, *P* < 0.001), 3-year overall survival rate (82% vs. 46%) and 5-year overall survival rate (63% vs. 30%) were also favorable. Regimens used for neoadjuvant therapy included FOLFIRINOX in 45.3% and GnP in 28.5%, with 50.0% of cases receiving concomitant radiotherapy. Both conventional and stereotactic radiotherapy were independent factors associated with pCR. However, Stoop et al. also noted that 5-year relapse-free survival rate of pCR patients was 64%, suggesting possible recurrence even in pCR patients. Therefore, it remains controversial whether pCR can be considered a definitive cure, as there may still be micrometastasis present even in pCR patients. In Case 1, pCR was achieved without radiotherapy, but solely with two courses of neoadjuvant GS therapy, indicating an extremely high drug sensitivity of the tumor. The patient has remained free of recurrence to date, but careful follow-up is necessary in this case as well.

Despite pStage I, Case 2 experienced early postoperative liver metastasis resulting in extremely poor prognosis. One possible reason was the presence of pathological splenic vein involvement. The splenic vein is the main drainage vessel of the pancreatic body and tail, directly draining into the portal vein. Splenic vein involvement in pancreatic cancer is considered to be a poor prognostic factor and risk factor for liver metastasis. In a systematic review of patients undergoing distal pancreatectomy for resectable pancreatic body and tail cancer, Crippa et al. reported that cases with pathological splenic vein involvement had significantly shorter survival compared with those without.^12)^ Furthermore, Mizumoto et al. showed that in resectable cases, both pathological and radiological splenic vein involvement were independent risk factors for early liver metastasis recurrence, with a median time to recurrence of 5.1 months.^13)^ On the other hand, evidence is limited regarding the correlation between pathological splenic vein involvement and prognosis in patients with resectable pancreatic cancer after neoadjuvant therapy. Takahashi et al. showed that radiological splenic artery invasion was an independent factor for poor prognosis, but the relationship between pathological splenic vein involvement and prognosis was unclear.^14)^ The concept of neoadjuvant therapy is to control micrometastasis early after diagnosis. However, as in our case, pathological splenic vein involvement may be a powerful negative prognostic factor even under neoadjuvant GS therapy. Further study is needed to address whether neoadjuvant GS therapy is appropriate to prolong survival of patients with splenic vein involvement.

In Case 2, it is possible that there was micrometastasis in the liver that could not be detected by imaging before surgery. If detected, aggressive systemic chemotherapy regimens could have been used and unnecessary surgery avoided. Currently, liquid biopsy including the analysis of tumor-derived DNA (circulating tumor DNA), circulating tumor cells, and exosomes secreted by cancer cells in blood samples^15)^ are in development for detecting potential micrometastasis. For example, Hata et al. reported that circulating tumor DNA detecting KRAS gene codon 12/13 mutations is an effective predictive marker for occult pancreatic cancer metastasis.^16)^ In future, if the detection of micrometastasis in pancreatic cancer becomes feasible, strategies such as controlling micrometastasis before attempting resection could be considered, leading to further improvements in the prognosis of pancreatic cancer patients.

Cases 1 and 2 had similar tumor characteristics, treatment courses, and perioperative outcomes, yet their prognoses differed significantly. Tumor biology likely played a key role, as differences in gemcitabine sensitivity and resistance are well known. hENT1 and dCK expression are associated with improved gemcitabine efficacy and survival,^17)^ while RRM1 expression is related to acquired drug resistance.^18,19)^ Although this study did not analyze hENT1, dCK, or RRM1, their presence or absence may have influenced outcomes.

## CONCLUSIONS

Two cases of resectable pancreatic cancer following neoadjuvant GS therapy had opposite outcomes: One achieved a pCR and has remained recurrence-free, while the other experienced early recurrence after surgery with rapid progression to death. Although very rare, neoadjuvant GS therapy has potential to yield pCR. Nevertheless, even after surgical resection, some patients still exhibit an extremely poor prognosis. Therefore, it is necessary to clarify their clinical characteristics.

## ACKNOWLEDGEMENTS

We sincerely appreciate Dr. Kae Fujinuma at the Department of Surgery, Jichi Medical University School of Medicine for data collection and data analysis.

## DECLARATIONS

### Funding

None.

### Authors’ contributions

MT is the first author and drafted the manuscript.

HS performed a critical review of the manuscript.

MT and MS conducted data collection and analysis.

HM and HO administered chemotherapy to these patients.

HS, YM, MT, and KM performed the operations in these cases.

NS, HK, and NF conducted histopathological diagnoses.

All authors reviewed and revised the manuscript.

All authors read and approved the final version of the manuscript.

### Availability of data and materials

Not applicable.

### Ethics approval and consent to participate

This study was approved by the Central Ethics Committee for Clinical Research of Jichi Medical University (A21-064).

### Consent for publication

This study was approved by the Central Ethics Committee for Clinical Research of Jichi Medical University (A21-064). The patients provided permissions to publish their cases.

### Competing interests

The authors declare that they have no competing interests.
